# Optimizing the lysis step in CTAB DNA extractions of silica‐dried and herbarium leaf tissues

**DOI:** 10.1002/aps3.11522

**Published:** 2023-05-27

**Authors:** S. James Carey, L. Ellie Becklund, Paige P. Fabre, John J. Schenk

**Affiliations:** ^1^ Department of Environmental and Plant Biology Ohio University Athens Ohio 45701‐2979 USA

**Keywords:** cell lysis, cetyltrimethylammonium bromide, CTAB, DNA extraction, molecular biology, plant genetics

## Abstract

**Premise:**

The use of cetyltrimethylammonium bromide (CTAB) is an effective and inexpensive method of extracting DNA from plants. The CTAB protocol is frequently modified to optimize DNA extractions, but experimental approaches rarely perturb a single variable at a time to systematically infer their effect on DNA quantity and quality.

**Methods and Results:**

We investigated how chemical additives, incubation temperature, and lysis duration affected DNA quantity and quality. Altering those parameters influenced DNA concentrations and fragment lengths, but only extractant purity was significantly affected. CTAB and CTAB plus polyvinylpyrrolidone buffers produced the highest DNA quality and quantity. Extractions from silica gel–preserved tissues had significantly higher DNA yield, longer DNA fragments, and purer extractants compared to herbarium‐preserved tissues.

**Conclusions:**

We recommend DNA extractions of silica gel–preserved tissues that include a shorter and cooler lysis step, which results in purer extractions compared to a longer and hotter lysis step, while preventing fragmentation and reducing time.

Efficiently extracting large quantities of high‐quality DNA from a diversity of species is crucial to the study of genetics and evolution. DNA is most commonly extracted from plant tissues using cetyltrimethylammonium bromide (CTAB), a detergent used to lyse cells and release genomic DNA from the protoplast. The Doyle and Doyle (1987) CTAB protocol publicized its efficiency and versatility across species and detailed numerous modifications to optimize extractions across plant groups. Although many researchers have since modified the CTAB protocol individually to best optimize DNA extractions in their groups (Schenk et al., 2023), surprisingly few studies have been published that experimentally optimize reactions.

Due to the unique composition of secondary metabolites across plant species, the universal success of a single CTAB protocol for all species is unlikely (Doyle and Doyle, 1987), which has resulted in customized protocols that include additional salts, detergents, polymers, and reducing agents to optimize DNA extractions in different taxonomic groups (e.g., Maguire et al., 1994; Michiels et al., 2003). Plant DNA extractions present two major problems: oxidation caused by phenolic compounds and coprecipitation of polysaccharides. Oxidizing phenolic compounds covalently bond to DNA and are subsequently lost in the isolation step (Loomis and Battaile, 1966; Japelaghi et al., 2011), resulting in low‐yield extractions. Oxidation, which can be observed by the brown‐pigmentated isolate, is caused by quinones that polymerize during a phenoloxidase reaction caused by endogenous phenolics that react when the tissue is macerated (Anderson, 1968). Polysaccharide coprecipitation is problematic because it can affect downstream molecular applications and can make the lysate viscous, resulting in pipetting error (Shioda and Murakami‐Murofushi, 1987; Pandey et al., 1996; Varma et al., 2007).

To address extraction issues such as oxidation and coprecipitation of polysaccharides and phenolic compounds, additives and other detergents are introduced to CTAB extractions or, more rarely, are used in place of CTAB (e.g., Dellaporta et al., 1983; Scott and Playford, 1996; Ribeiro and Lovato, 2007). Three of the most common chemicals that are added to CTAB extractions, henceforth referred to as “additives,” are polyvinylpyrrolidone (PVP), sodium dodecyl sulfate (SDS), and sorbitol (Schenk et al., 2023). PVP is an inert polymer that bonds to phenolic compounds, creating high‐molecular‐weight molecules that are subsequently removed from the extractant (Loomis and Battaile, 1966; John, 1992). PVP also prevents oxidation by suppressing the formation of quinones that would otherwise compromise DNA quality and quantity (Loomis and Battaile, 1966; Rogers and Bendich, 1985; Doyle and Doyle, 1990; Nazhad and Solouki, 2008; Schenk et al., 2023). Three forms of PVP are added to CTAB extractions, including PVP‐40, PVP‐10 (a lower‐molecular‐weight version), and polyvinylpolypyrrolidone (PVPP), an insoluble cross‐linked version of PVP (Porebski et al., 1997; Schenk et al., 2023). SDS is a detergent that breaks apart cell components and bonds with proteins and polysaccharides to facilitate their removal (Kasem et al., 2008; Ramachandran et al., 2022; Schenk et al., 2023). Sorbitol, a reducing agent, is an additive that can be used at high concentrations to reduce oxidation (Stein, 1993; Kasem et al., 2008). Although additives are commonly applied during the extraction process, a lack of comparative experiments makes it difficult to interpret their efficacy.

In addition to the use of chemical additives, the eight individual steps of the standard CTAB protocol are modified across labs and study systems, and consist of tissue preparation, suspension, lysis, isolation, cleaning, elution, secondary cleanup, and quantification (Schenk et al., 2023). Experimentally testing how each step of the extraction process can be optimized could lead to more effective DNA extractions across groups, but doing so is unfeasible in a single publication. Optimization of the lysis step, however, is critical because it is when oxidation occurs (Csaikl et al., 1998). The lysis step involves macerating and incubating tissues with a detergent to lyse cells and is one of the most variable steps among published CTAB extraction protocols in the literature (Schenk et al., 2023). Not only does the chemical composition and concentration of lysis buffers vary across labs, but incubations also vary in temperature and duration, ranging between 55–70°C and 1–86,400 min across studies (Schenk et al., 2023). The optimal incubation duration and temperature have not been systematically determined in comparative studies that modify a single variable at a time.

The preservation method of plant tissues is another important consideration during DNA extractions (Drábková et al., 2002), as tissue preservation could influence optimal extraction conditions (e.g., silica‐dried vs. pressed). Although extracting from fresh or silica gel–preserved tissues is considered optimal for DNA extractions (Funk et al., 2017), in many studies it is not possible to sample live plant tissues and consequently it is necessary to use tissues from herbarium specimens that could vary in age and condition (e.g., Brewer et al., 2019). We do not know, however, how optimizing the lysis step impacts the quality and quantity of extracted DNA from silica‐dried and herbarium specimens.

Studies have been conducted testing different CTAB protocols against each other (Sahu et al., 2012; Arruda et al., 2017); however, while such studies are helpful for evaluating the protocols, their results are difficult to interpret because the protocols modified multiple conditions simultaneously, rather than perturbing a single condition at a time. To determine the most favorable conditions of DNA extractions, an experimental approach is needed to systematically test various conditions and objectively determine their effect. We first experimentally determined optimal incubation times and temperatures of four angiosperm groups (representing monocots, asterids, rosids, and Caryophyllidae) from tissues preserved as herbarium specimens and in silica gel desiccant. A second experiment used an exemplar that experienced high oxidation during the lysis step to determine whether CTAB protocols that included the additives PVP, SDS, or sorbitol produced higher‐yield extractions with less oxidation than a protocol without additives. We assessed quantity and quality by measuring DNA concentration, concentrations of coprecipitates, and DNA fragment lengths.

## METHODS

### Samples

Silica‐dried leaf material and herbarium specimens of four angiosperm genera were sampled to compare DNA quality and yield. Specimens were selected to represent major angiosperm clades and were of interest to our respective research programs (e.g., Schenk and Hufford, 2011; Schenk et al., 2018; Becklund and Ayers, 2022), which included monocots, Caryophyllidae, rosids, and asterids. Silica‐dried specimens included *Maianthemum racemosum* (L.) Link (Asparagaceae), *Paronychia sessiliflora* Nutt. (Caryophyllaceae), *Mentzelia decapetala* (Pursh) Urb. (Loasaceae), and *Vicia villosa* Roth (Fabaceae) (Appendix 1). Herbarium specimens included the same species as above except *Paronychia argyrocoma* (Michx.) Nutt. replaced *P. sessiliflora* because of tissue limitations in the latter species. Herbarium tissues were 16–60 (mean: 46.5) years old and silica‐dried tissues were 0–18 (mean: 4.75) years old (Appendix 1). For the additive experiment, only the silica‐dried *M. decapetala* was tested to determine whether oxidation could be reduced with the use of additives (Appendix S1).

### Incubation experiment

Studies have suggested that lower temperatures (<60°C) might reduce oxidation (Arruda et al., 2017), which led us to explore how altering incubation temperature and duration could affect DNA yield and quality. We conducted experiments that perturbed either temperatures or incubation duration and compared the DNA quality and quantity of the extractants.

#### DNA extractions

We applied the CTAB DNA extraction protocol as detailed in Schenk et al. (2023, their Appendix S1) with the amendments described here and detailed in Appendix S2. Twelve replicates of leaf tissue from each sample (*N*_species/silica_ = 4, *N*_species/herbarium_ = 4) were weighed according to the amount of material available, which was less for herbarium (~5.0 mg) than silica‐dried (8.0–10.0 mg) specimens. Dry material was ground in a Fisherbrand Bead Mill 24 Homogenizer (Thermo Fisher Scientific, Waltham, Massachusetts, USA) for 2–4 min, with the longer grinding times used as needed to assure that the leaf material was finely ground. After homogenization, replicated lysates rested at room temperature for 15–20 min with the tube caps loosened to reduce foam and maximize the usable liquid volume. Lysates from the 12 replicates per species were pooled and mixed by pipetting to standardize DNA concentrations of starting material per replicate, then 400 µL of the pooled homogenate was aliquoted to 12 1.5‐mL microcentrifuge tubes. Replicates were randomly assigned to one of the four incubation treatments (50°C, 55°C, 60°C, and 65°C) and three duration treatments (1 h, 2 h, and “overnight” [18–20 h]). The temperatures and durations were based on commonly applied values in the literature as assessed by Schenk et al. (2023).

#### Quantification

DNA concentrations of each replicate (96 total) were measured using the Qubit 4 Fluorometer (Thermo Fisher Scientific). DNA fragment sizes between 100–60,000 bp were quantified using the Agilent TapeStation (Agilent, Santa Clara, California, USA). DNA extractant quality was estimated using the NanoDrop (Thermo Fisher Scientific), and its purity assessed by the A_260_/A_280_ and A_260_/A_230_ UV absorbance ratios. DNA absorbs light at A_260_, while secondary metabolites like polysaccharides and phenolic compounds absorb light at A_230_. The A_260_/A_230_ ratio, therefore, measures the ratio of DNA to secondary metabolites. Likewise, the A_260_/A_280_ ratio measures the ratio of DNA to protein (A_280_) contamination. We tested for significant differences in treatments with an analysis of covariance in R (R Core Team, 2005). We standardized our measurement of DNA yield by estimating the total amount of DNA in the extractant given the starting tissue amount, and all data were log‐transformed to approximate normality.

### Additive experiment

A second experiment tested the effect of three additives—PVP‐10, SDS, and sorbitol (see Schenk et al., 2023, their Appendices [Link], [Link])—compared to control extractions using CTAB only. For this experiment, we used only *M. decapetala* because it experienced considerable oxidation compared to the other three species. We still followed the CTAB DNA extraction protocol as detailed in Schenk et al. (2023, their Appendix S1), but with the following amendments to Extraction Steps 1–3.

For all treatments, silica gel–preserved leaves of two replicates were weighed to ~10.0 mg and ground in a Fisherbrand Bead Mill 24 Homogenizer for 2 min (1 min dry, 1 min with buffer). For the SDS, PVP‐10, and CTAB‐only treatments, the replicates were ground in a CTAB and β‐mercaptoethanol solution, pooled, and 360 μL of homogenate were aliquoted to two 1.5‐mL microcentrifuge tubes. Four microliters each of 10% SDS, 0.2% PVP‐10, and distilled water (in CTAB‐only to maintain identical total extraction volumes across treatments) were added to their respective treatments. For the sorbitol treatment (Štorchová et al., 2000), the following additional adjustments were required as sorbitol is a suspension buffer that must be added prior to CTAB. After the dry tissue grind, 1.3 mL of 1% sorbitol buffer was added to each replicate, mixed by pipetting, and left at room temperature for 15 min to allow the polyphenols to suspend in the buffer. The replicates were centrifuged at 14,000 rpm for 8 min, and the supernatant was removed. An additional 211.29 μL of 1% sorbitol buffer, 3 μL of β‐mercaptoethanol, and 285.71 μL of CTAB buffer were added to each replicate (for a total of 500 μL of lysate buffer across treatments) and then ground for an additional minute. The replicates were pooled and 360 μL was aliquoted into individual replicates as described for the above treatments, followed by 4 μL of distilled water to bring the total volume to 364 μL. All remaining extraction steps were identical across treatments. The samples were quantified as stated above in the incubation‐experiment methods, and the significance was assessed with an analysis of covariance of log‐transformed data in R.

## RESULTS

**Figure 1:**
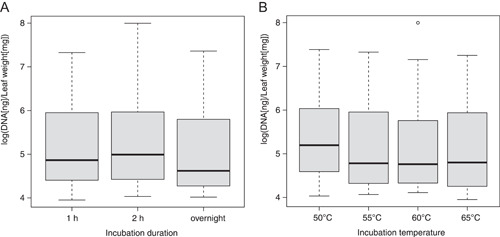
Boxplot results of the incubation experiment to determine the effects of incubation (A) duration and (B) temperature on the amount of total DNA (in nanograms) per input tissue weight (in milligrams). An analysis of covariance determined there to be no significant difference.

**Figure 2:**
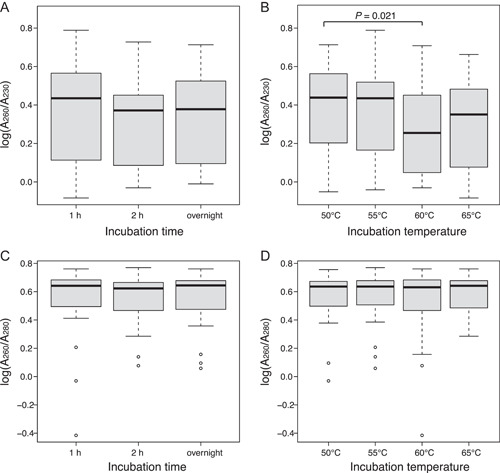
Boxplot results of the incubation experiment to determine the effects of incubation (A, C) duration and (B, D) temperature on the ratio of the (A, B) A_260_ (=DNA) and A_230_ (=secondary metabolites), and the (C, D) A_260_ (=DNA) and A_280_ (=proteins) spectra. An analysis of covariance determined there to be a significantly higher A_260_/A_230_ ratio for cooler temperatures (*P* = 0.021); all other values were not significantly different.

**Figure 3:**
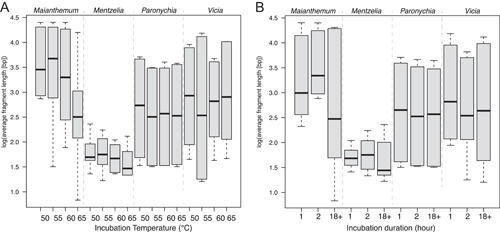
Boxplot results of the incubation experiment displaying the log‐transformed DNA fragment sizes and incubation (A) temperature and (B) duration for each species.

**Figure 4:**
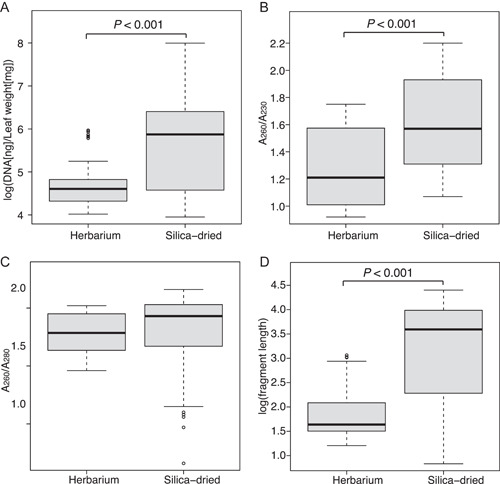
Boxplot results of the incubation experiment that demonstrated that herbarium samples produced (A) less DNA per tissue weight, (B) lower A_260_/A_230_ ratio, (C) lower A_260_/A_280_ ratio, and (D) smaller fragments. An analysis of covariance determined there to be significant differences between herbarium and silica gel–preserved tissues in yield (*P* < 0.001), A_260_/A_230_ ratio (*P* < 0.001), and fragment length (*P* < 0.001).

Although shorter and cooler incubation times appeared to result in higher yields on average (Figure 1, Appendix S3), the results were not significantly different than longer and warmer incubations (*P* = 0.303, *F*_2,93_ = 1.210 vs. *P* = 0.252, *F*_3,92_ = 1.389, respectively). Cooler temperatures resulted in significantly less secondary metabolite carryover as measured with the A_260_/A_230_ ratios (*P* = 0.021, *F*_3,92_ = 3.419; Figure 2), but there was not a significant difference when comparing duration. DNA fragments averaged 3080 bp across all treatments but ranged from 647–8614 bp (Appendix S3). Lower incubation temperatures and shorter durations tended to result in longer DNA fragments as measured with a fragment analyzer, but the difference in DNA integrity number (DIN; a measure of fragmentation across the size classes, where lower numbers indicate more degraded DNA) values was not significant (*P* = 0.227, *F*_3,92_ = 1.476 vs. *P* = 0.180, *F*_2,93_ = 1.752, respectively; Figure 3). The A_260_/A_280_ ratios remained indistinguishable across all trials. Cooler temperatures did not reduce oxidation, as quantified by extractant pigmentation. Compared across species, DNA extractions resulted in significantly different amounts of total DNA yield extracted per tissue weight (*P* < 0.001, *F*_3,92_ = 52.711; Appendices S3–S5). DNA extracted from silica gel–preserved leaves had significantly higher yields (*P* < 0.001, *F*_1,94_ = 85.898; Figure 4A), less fragmentation (*P* < 0.001, *F*_1,94_ = 156.596; Figure 4D), and contained fewer secondary metabolites (*P* < 0.001, *F*_1,94_ = 65.255; Figure 4B) compared to herbarium specimens, but protein carryover was not different (Figure 4C).

The additive experiment determined that the addition of PVP‐10 produced the highest yields of DNA (Table 1), closely followed by CTAB without additives. The addition of sorbitol resulted in the lowest yields, but produced the longest average fragment size, albeit with the lowest estimated DIN (Table 1). Significant differences were identified in DNA yield (*P* < 0.001, *F*_3,4_ = 25.327), with sorbitol being significantly lower than CTAB, PVP, and SDS; in A_260_/A_230_ ratios (*P* < 0.001, *F*_3,4_ = 117.400), with sorbitol having higher relative secondary metabolites than CTAB, PVP, and SDS; and in A_260_/A_280_ ratios (*P* = 0.016, *F*_3,4_ = 12.681), with PVP having more relative protein carryover than SDS and sorbitol. However, average fragment lengths were not significantly different (*P* = 0.361, *F*_3,4_ = 1.417).

**Table 1:** DNA yield and quality values from the additive CTAB experiment with *Mentzelia decapetala*. All values are averages based on two replicates.

Additive	Concentration (ng/μL)	Total DNA/leaf weight (mg)	A_260_/A_230_ ratio^a^	A_260_/A_280_ ratio^a^	Length (bp)^b^	DIN^c^
PVP‐10	14.8	74	1.21	1.52	1770.5	1.55
SDS	5.47	27.33	0.85	1.66	2209.5	1.7
Sorbitol	1.71	28.85	0.38	1.67	3237	1.3
None	9.35	46.75	0.93	1.56	1966.5	1.7

*Note*: DIN = DNA integrity number, PVP‐10 = polyvinylpyrrolidone (lower molecular weight), SDS = sodium dodecyl sulfate.

^a^ A_260_/A_230_ is a ratio of DNA to secondary metabolites, and A_260_/A_280_ is a ratio of DNA to proteins.

^b^ DNA fragment length.

^c^ DIN measures fragmentation across size classes, where lower numbers indicate more degraded DNA.

## DISCUSSION

DNA extractions with CTAB are widely successful across a diversity of plant species, but they have been continuously modified to account for varying plant tissue compositions. Although many of these modifications have been documented and published (Schenk et al., 2023), studies that directly compare protocol modifications are lacking. We extracted DNA from both herbarium and silica‐dried specimens across four genera, altering the lysis step to include differing combinations of four incubation temperatures and three incubation times. We concluded that cooler temperatures and shorter incubations resulted in at least equal yields of DNA extractants with less fragmentation and contamination compared to warmer temperatures and longer incubation times. Although, on average, shorter and cooler incubations resulted in greater DNA yield, differences in yield were not statistically significant; however, this might reflect our low replicate number rather than the magnitude of the impact.

In comparisons among different species, some species (e.g., *M. racemosum*) had less fragmentation than others at the same temperatures and durations (Appendices S4, S5). For downstream applications, such as constructing genomic libraries, additional fragmentation may not be necessary if the average length is <500 bp (Hale et al., 2020). Regardless, we recommend avoiding DNA degradation resulting from extraction temperature or duration because high‐quality DNA at high concentrations better facilitates successful library preparation and sequencing, and controlling fragmentation through sonication or enzymatic reactions should produce more desirable size distribution results.

Our additive experiment tested the addition of PVP‐10, SDS, and sorbitol on the quality and yield of DNA extractions. This experiment was performed on *M. decapetala* due to its relatively high level of oxidation in our incubation experiment (Appendix S1). Oxidation was still observed in all four treatments, regardless of the chemical additive used. The sorbitol reaction produced the lightest‐colored lysate (Appendix S1), but that was likely due to the lysate first being subjected to a suspension buffer that was later diluted, rather than a reduction of oxidation. Carryover of secondary metabolites, as estimated with the A_260_/A_230_ ratio, was most successfully prevented by the addition of PVP‐10 (Table 1). PVP‐10 extractions also yielded the highest DNA concentrations (Table 1), which is consistent with the findings of Nazhad and Solouki (2008), who also produced greater DNA concentrations when PVP was added. SDS extractions yielded less DNA compared to extractions with PVP‐10 or no additive, but also resulted in high secondary metabolite carryover (Table 1). If *M. decapetala* is representative of other plant species, we do not recommend the addition of SDS in the lysis buffer unless excessive polysaccharides are present in the leaf tissues; however, studies on additional species are needed to determine if this is generally true. Sorbitol extractions yielded the lowest concentration of DNA with the poorest A_260_/A_230_ ratio (Table 1); however, they did result in the longest fragments (Table 1), which may be appealing for long‐read sequencing.

We conclude with several recommendations based on the results of this study. First, we recommend that incubation duration does not exceed one hour. Longer incubation times on average resulted in lower yield, smaller DNA fragments, and greater contamination, and although these differences were not significant, they demonstrate that longer incubations are no more efficient than the 1‐h incubation. Second, we recommend that incubation temperatures be kept around 50–55°C and not exceed 60°C. Hotter temperatures resulted in shorter DNA fragments when comparing means (although differences were not statistically significant), whereas cooler temperatures resulted in higher yields and higher A_260_/A_230_ ratios (with only the latter being significant). Third, we recommend that recent silica‐dried tissue be used instead of tissue from herbarium specimens whenever possible. DNA extracted from silica‐dried tissue on average had significantly higher DNA concentrations, longer average DNA fragment lengths, and higher A_260_/A_230_ and A_260_/A_280_ ratios than DNA extracted from herbarium specimens. Although no single DNA extraction protocol can be universally successful, the results of comparative studies, such as ours, can reveal best practices for addressing issues that are known to compromise extraction success.

## AUTHOR CONTRIBUTIONS

This project was envisioned and designed by L.E.B. and J.J.S., experiments were conducted by S.J.C. and P.P.F., and the manuscript was written by S.J.C., J.J.S., L.E.B., and P.P.F. All authors approved the final version of the manuscript.

## Supporting information


**Appendix S1.** Lysate from additive experiment of *Mentzelia decapetala* (Loasaceae) that has oxidized. Two replicates of each treatment are included and have been photographed at the completion of the lysis step. PVP = polyvinylpyrrolidone, SDS = sodium dodecyl sulphate, CTAB = cetyltrimethylammonium bromide.

**Appendix S2.** Protocol for optimizing the lysis step in CTAB DNA extractions of silica‐dried and herbarium leaf tissues, based on Schenk et al. (2023).

**Appendix S3.** Results from the incubation experiment.

**Appendix S4.** Effect of differences in incubation duration for each of the four taxonomic groups on the (A) A_260_/A_230_ ratio, (B) A_260_/A_280_ ratio, (C) log‐transformed average fragment length, and (D) log‐transformed total amount of DNA given the input leaf tissue weight.

**Appendix S5.** Effect of differences in incubation temperature for each of the four taxonomic groups on the (A) A_260_/A_230_ ratio, (B) A_260_/A_280_ ratio, (C) log‐transformed average fragment length, and (D) log‐transformed total amount of DNA given the input leaf tissue weight.

## Data Availability

All data are published in the manuscript and Supporting Information.

## APPENDIX 1

List of specimens used in this study with their associated voucher numbers. Herbarium acronyms are according to Index Herbariorum (Thiers, 2023).

*Maianthemum racemosum* (L.) Link (Asparagaceae), silica gel: *Becklund 80*, 27 May 2022 (BHO), herbarium material: *Butala 193*, 15 May 1965 (BHO); *Paronychia sessiliflora* Nutt. (Caryophyllaceae), silica gel: *Schenk 2636*, 6 July 2021 (GAS), herbarium material (as *Paronychia argyrocoma* (Michx.) Nutt.): *Bartley s.n*., 25 June 1969 (BHO); *Mentzelia decapetala* (Pursh) Urb. (Loasaceae), silica gel: *Schenk 939*, 23 July 2004 (WS), herbarium material: *Schenk 1808*, 26 July 2006 (WS); and *Vicia villosa* Roth (Fabaceae), silica gel: *Becklund 79*, 26 May 2022 (BHO), herbarium material: *Murphrey s.n*., 4 October 1962 (BHO).
