# Supplementary material for: Optimizing the lysis step in CTAB DNA extractions of silica‐dried and herbarium leaf tissues
Source: Appl Plant Sci. 2023 May 27;11(3):e11522. doi: 10.1002/aps3.11522 (PMC10278933; doi:10.1002/aps3.11522)

DOI 10.1002/aps3.11522

APPENDIX S1. Lysate from additive experiment of *Mentzelia decapetala* (Loasaceae) that has oxidized. Two replicates of each treatment are included and have been photographed at the completion of the lysis step. PVP = polyvinylpyrrolidone, SDS = sodium dodecyl sulphate, CTAB = cetyltrimethylammonium bromide.

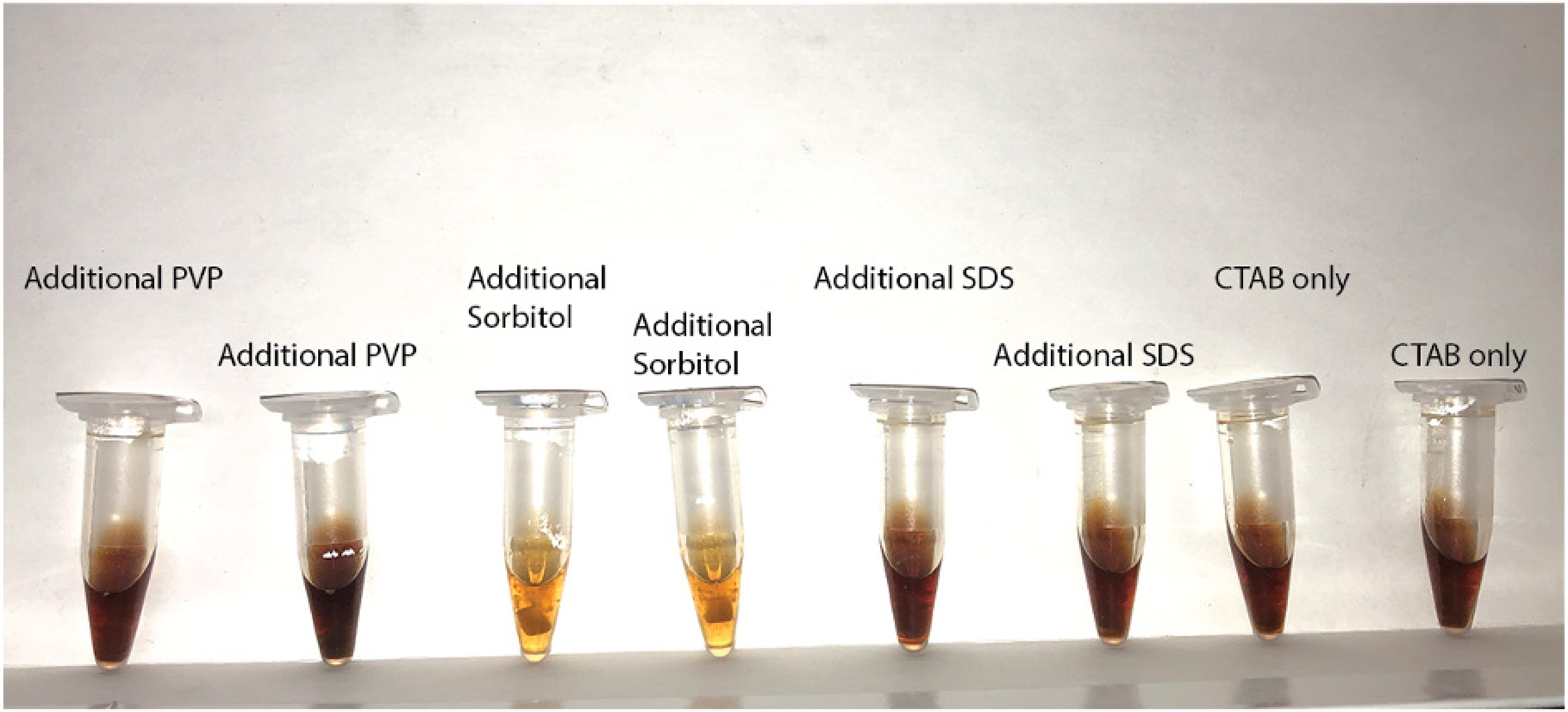

Supplement: Supplementary file 1 — Appendix S1. Lysate from additive experiment of Mentzelia decapetala (Loasaceae) that has oxidized. Two replicates of each treatment are included and have been photographed at the completion of the lysis step. PVP = polyvinylpyrrolidone, SDS = sodium dodecyl sulphate, CTAB = cetyltrimethylammonium bromide. [file APS3-11-e11522-s005.pdf]
