# Supplementary material for: Optimizing the lysis step in CTAB DNA extractions of silica‐dried and herbarium leaf tissues
Source: Appl Plant Sci. 2023 May 27;11(3):e11522. doi: 10.1002/aps3.11522 (PMC10278933; doi:10.1002/aps3.11522)
Supplement: Supplementary file 3 — Appendix S3. Results from the incubation experiment. [file APS3-11-e11522-s004.docx]

**Appendix S3.** Results from the incubation experiment.

| **Species** | **Type**^a^ | **Incubation time (h)** | **Incubation temp. (°C)** | **Tissue weight (mg)** | **Conc. (ng/μL)^b^** | **DNA/weight^c^** | **260/230^d^** | **260/280^e^** | **Length^f^** | **DIN^g^** |
| --- | --- | --- | --- | --- | --- | --- | --- | --- | --- | --- |
| *Maianthemum racemosum* | Herb. | 1 | 50 | 5.1 | 38.5 | 377.5 | 1.63 | 1.89 | 690 | 1 |
| *Maianthemum racemosum* | Herb. | 1 | 55 | 5.1 | 39.9 | 391.2 | 1.56 | 1.86 | 647 | 1 |
| *Maianthemum racemosum* | Herb. | 1 | 60 | 5.1 | 11.7 | 114.7 | 1.39 | 1.99 | 1094 | 1.1 |
| *Maianthemum racemosum* | Herb. | 1 | 65 | 5.1 | 19.4 | 190.2 | 1.62 | 1.98 | 783 | 1 |
| *Maianthemum racemosum* | Herb. | 2 | 50 | 5.1 | 36 | 352.9 | 1.53 | 1.85 | 916 | 1 |
| *Maianthemum racemosum* | Herb. | 2 | 55 | 5.1 | 33.2 | 325.5 | 1.55 | 1.84 | 669 | 1 |
| *Maianthemum racemosum* | Herb. | 2 | 60 | 5.1 | 34.7 | 340.2 | 1.57 | 1.88 | 821 | 1 |
| *Maianthemum racemosum* | Herb. | 2 | 65 | 5.1 | 39.6 | 388.2 | 1.58 | 1.85 | 669 | 1 |
| *Maianthemum racemosum* | Herb. | 18–20 | 50 | 5.1 | 34.4 | 337.3 | 1.75 | 1.89 | 776 | 1 |
| *Maianthemum racemosum* | Herb. | 18–20 | 55 | 5.1 | 9.28 | 91 | 1.54 | 2.02 | 2040 | 1.2 |
| *Maianthemum racemosum* | Herb. | 18–20 | 60 | 5.1 | 6.3 | 61.8 | 0.99 | 1.97 | 1059 | 1 |
| *Maianthemum racemosum* | Herb. | 18–20 | 65 | 5.1 | 9.85 | 96.6 | 1.3 | 1.97 | 1037 | 1 |
| *Maianthemum racemosum* | Silica | 1 | 50 | 10.1 | 167 | 826.7 | 1.83 | 1.96 | 8152 | 6.3 |
| *Maianthemum racemosum* | Silica | 1 | 55 | 10.1 | 307 | 1519.8 | 1.57 | 1.95 | 7464 | 6 |
| *Maianthemum racemosum* | Silica | 1 | 60 | 10.1 | 245 | 1212.9 | 1.79 | 1.99 | 8300 | 6.1 |
| *Maianthemum racemosum* | Silica | 1 | 65 | 10.1 | 68.3 | 338.1 | 1.76 | 2 | 7577 | 6 |
| *Maianthemum racemosum* | Silica | 2 | 50 | 10.1 | 326 | 1613.9 | 1.72 | 1.94 | 7382 | 6.1 |
| *Maianthemum racemosum* | Silica | 2 | 55 | 10.1 | 291 | 1440.6 | 1.54 | 1.96 | 7436 | 6.1 |
| *Maianthemum racemosum* | Silica | 2 | 60 | 10.1 | 600 | 2970.3 | 1.47 | 1.98 | 8614 | 6.1 |
| *Maianthemum racemosum* | Silica | 2 | 65 | 10.1 | 285 | 1410.9 | 1.45 | 1.95 | 8600 | 6.2 |
| *Maianthemum racemosum* | Silica | 18–20 | 50 | 10.1 | 318 | 1574.3 | 1.76 | 1.97 | 6333 | 5.8 |
| *Maianthemum racemosum* | Silica | 18–20 | 55 | 10.1 | 302 | 1495 | 1.68 | 1.99 | 6698 | 5.8 |
| *Maianthemum racemosum* | Silica | 18–20 | 60 | 10.1 | 259 | 1282.2 | 1.51 | 1.97 | 6507 | 5.7 |
| *Maianthemum racemosum* | Silica | 18–20 | 65 | 10.1 | 216 | 1069.3 | 1.42 | 1.97 | 6321 | 5.7 |
| *Mentzelia decapetala* | Herb. | 1 | 50 | 5.1 | 7.51 | 73.6 | 0.95 | 1.63 | 2862 | 1.1 |
| *Mentzelia decapetala* | Herb. | 1 | 55 | 5.1 | 7.53 | 73.8 | 0.96 | 1.64 | 2808 | 1.9 |
| *Mentzelia decapetala* | Herb. | 1 | 60 | 5.1 | 7.87 | 77.2 | 0.97 | 1.64 | 2525 | 2 |
| *Mentzelia decapetala* | Herb. | 1 | 65 | 5.1 | 6.82 | 66.9 | 0.92 | 1.65 | 2793 | 2.1 |
| *Mentzelia decapetala* | Herb. | 2 | 50 | 5.1 | 8.96 | 87.8 | 1.02 | 1.66 | 2735 | 1.3 |
| *Mentzelia decapetala* | Herb. | 2 | 55 | 5.1 | 7.74 | 75.9 | 1.01 | 1.68 | 2655 | 2 |
| *Mentzelia decapetala* | Herb. | 2 | 60 | 5.1 | 7.76 | 76.1 | 1.01 | 1.59 | 2741 | 2 |
| *Mentzelia decapetala* | Herb. | 2 | 65 | 5.1 | 6.37 | 62.5 | 0.97 | 1.71 | 2887 | 2 |
| *Mentzelia decapetala* | Herb. | 18–20 | 50 | 5.1 | 8.96 | 87.8 | 1 | 1.73 | 2595 | 1.4 |
| *Mentzelia decapetala* | Herb. | 18–20 | 55 | 5.1 | 8.74 | 85.7 | 1.02 | 1.68 | 2484 | 1.7 |
| *Mentzelia decapetala* | Herb. | 18–20 | 60 | 5.1 | 6.59 | 64.6 | 1.02 | 1.68 | 2746 | 1.7 |
| *Mentzelia decapetala* | Herb. | 18–20 | 65 | 5.1 | 7.18 | 70.4 | 1.01 | 1.69 | 2748 | 2.1 |
| *Mentzelia decapetala* | Silica | 1 | 50 | 10.2 | 15.6 | 76.5 | NA | 0.97 | 1760 | 1.5 |
| *Mentzelia decapetala* | Silica | 1 | 55 | 10.2 | 18.9 | 92.6 | 2.2 | 1.23 | 1872 | 1.5 |
| *Mentzelia decapetala* | Silica | 1 | 60 | 10.2 | 15.8 | 77.5 | NA | 0.66 | 1868 | 1.5 |
| *Mentzelia decapetala* | Silica | 1 | 65 | 10.2 | 10.6 | 52 | 1.38 | 1.51 | 1454 | 1.4 |
| *Mentzelia decapetala* | Silica | 2 | 50 | 10.2 | 11.5 | 56.4 | 1.37 | 1.52 | 1311 | 1.4 |
| *Mentzelia decapetala* | Silica | 2 | 55 | 10.2 | 13.3 | 65.2 | NA | 1.15 | 1586 | 1.5 |
| *Mentzelia decapetala* | Silica | 2 | 60 | 10.2 | 15 | 73.5 | NA | 1.08 | 1927 | 1.4 |
| *Mentzelia decapetala* | Silica | 2 | 65 | 10.2 | 16.2 | 79.4 | NA | 1.33 | 1338 | 1.5 |
| *Mentzelia decapetala* | Silica | 18–20 | 50 | 10.2 | 20.8 | 102 | 2.04 | 1.1 | 1200 | 1.4 |
| *Mentzelia decapetala* | Silica | 18–20 | 55 | 10.2 | 14.9 | 73 | NA | 1.06 | 1676 | 1.3 |
| *Mentzelia decapetala* | Silica | 18–20 | 60 | 10.2 | 15 | 73.5 | NA | 1.17 | 1570 | 1.5 |
| *Mentzelia decapetala* | Silica | 18–20 | 65 | 10.2 | 11.5 | 56.4 | 1.5 | 1.43 | 1986 | 1.5 |
| *Paronychia argyrocoma* | Herb. | 1 | 50 | 5.2 | 10 | 96.2 | 1.12 | 1.66 | 1393 | 1.1 |
| *Paronychia argyrocoma* | Herb. | 1 | 55 | 5.2 | 7.77 | 74.7 | 1.06 | 1.71 | 1697 | 1.1 |
| *Paronychia argyrocoma* | Herb. | 1 | 60 | 5.2 | 10.6 | 101.9 | 1.01 | 1.57 | 1910 | 1.4 |
| *Paronychia argyrocoma* | Herb. | 1 | 65 | 5.2 | 9 | 86.5 | 0.98 | 1.6 | 1848 | 1.2 |
| *Paronychia argyrocoma* | Herb. | 2 | 50 | 5.2 | 10.5 | 101 | 1.07 | 1.57 | 1921 | 1.3 |
| *Paronychia argyrocoma* | Herb. | 2 | 55 | 5.2 | 10.3 | 99 | 1.04 | 1.58 | 1570 | 1.1 |
| *Paronychia argyrocoma* | Herb. | 2 | 60 | 5.2 | 10.7 | 102.9 | 1.05 | 1.6 | 1717 | 1.2 |
| *Paronychia argyrocoma* | Herb. | 2 | 65 | 5.2 | 11.4 | 109.6 | 1.09 | 1.58 | 1712 | 1.3 |
| *Paronychia argyrocoma* | Herb. | 18–20 | 50 | 5.2 | 11.9 | 114.4 | 1.2 | 1.46 | 1606 | 1.3 |
| *Paronychia argyrocoma* | Herb. | 18–20 | 55 | 5.2 | 10.5 | 101 | 1.32 | 1.47 | 1741 | 1.1 |
| *Paronychia argyrocoma* | Herb. | 18–20 | 60 | 5.2 | 7.87 | 75.7 | 1.1 | 1.49 | 1540 | 1.1 |
| *Paronychia argyrocoma* | Herb. | 18–20 | 65 | 5.2 | 7.33 | 70.5 | 1 | 1.54 | 1550 | 1.2 |
| *Paronychia sessiflora* | Silica | 1 | 50 | 8.1 | 67.5 | 416.7 | 1.37 | 1.89 | 2601 | 2.1 |
| *Paronychia sessiflora* | Silica | 1 | 55 | 8.1 | 76.4 | 471.6 | 1.3 | 1.92 | 3676 | 2.2 |
| *Paronychia sessiflora* | Silica | 1 | 60 | 8.1 | 56.5 | 348.8 | 1.29 | 1.91 | 4302 | 2.2 |
| *Paronychia sessiflora* | Silica | 1 | 65 | 8.1 | 60.1 | 371 | 1.26 | 1.88 | 4036 | 2.5 |
| *Paronychia sessiflora* | Silica | 2 | 50 | 8.1 | 68 | 419.8 | 1.31 | 1.89 | 2581 | 1.9 |
| *Paronychia sessiflora* | Silica | 2 | 55 | 8.1 | 67.5 | 416.7 | 1.26 | 1.88 | 3616 | 2.1 |
| *Paronychia sessiflora* | Silica | 2 | 60 | 8.1 | 50.4 | 311.1 | 1.23 | 1.88 | 4522 | 2.5 |
| *Paronychia sessiflora* | Silica | 2 | 65 | 8.1 | 63.7 | 393.2 | 1.21 | 1.89 | 3685 | 2 |
| *Paronychia sessiflora* | Silica | 18–20 | 50 | 8.1 | 60.8 | 375.3 | 1.25 | 1.88 | 2400 | 2.2 |
| *Paronychia sessiflora* | Silica | 18–20 | 55 | 8.1 | 61.8 | 381.5 | 1.18 | 1.9 | 3286 | 2.5 |
| *Paronychia sessiflora* | Silica | 18–20 | 60 | 8.1 | 52.3 | 322.8 | 1.16 | 1.82 | 3258 | 2.4 |
| *Paronychia sessiflora* | Silica | 18–20 | 65 | 8.1 | 48.9 | 301.9 | 1.07 | 1.91 | 2837 | 2.3 |
| *Vicia villosa* | Herb. | 1 | 50 | 5 | 10.6 | 106 | 1.6 | 1.98 | 1292 | 1 |
| *Vicia villosa* | Herb. | 1 | 55 | 5 | 14.1 | 141 | 1.61 | 1.95 | 1483 | 1 |
| *Vicia villosa* | Herb. | 1 | 60 | 5 | 11.9 | 119 | 1.53 | 1.95 | 991 | 1 |
| *Vicia villosa* | Herb. | 1 | 65 | 5 | 11.8 | 118 | 1.62 | 1.94 | 894 | 1 |
| *Vicia villosa* | Herb. | 2 | 50 | 5 | 12.4 | 124 | 1.55 | 1.94 | 1132 | 1 |
| *Vicia villosa* | Herb. | 2 | 55 | 5 | 17.4 | 174 | 1.6 | 1.98 | 1965 | 1 |
| *Vicia villosa* | Herb. | 2 | 60 | 5 | 13.6 | 136 | 1.22 | 1.85 | 837 | 1 |
| *Vicia villosa* | Herb. | 2 | 65 | 5 | 12.5 | 125 | 1.57 | 1.94 | 1008 | 1 |
| *Vicia villosa* | Herb. | 18–20 | 50 | 5 | 12.2 | 122 | 1.66 | 1.96 | 1677 | 1 |
| *Vicia villosa* | Herb. | 18–20 | 55 | 5 | 6.62 | 66.2 | 1.69 | 1.96 | 2206 | 1 |
| *Vicia villosa* | Herb. | 18–20 | 60 | 5 | 6.1 | 61 | 1.69 | 1.98 | 1233 | 1 |
| *Vicia villosa* | Herb. | 18–20 | 65 | 5 | 5.56 | 55.6 | 1.66 | 1.97 | 1375 | 1 |
| *Vicia villosa* | Silica | 1 | 50 | 10.1 | 112 | 554.5 | 1.94 | 2.07 | 5866 | 5.6 |
| *Vicia villosa* | Silica | 1 | 55 | 10.1 | 73 | 361.4 | 1.99 | 2.11 | 6651 | 5.8 |
| *Vicia villosa* | Silica | 1 | 60 | 10.1 | 30.3 | 150 | 2 | 2.14 | 6219 | 5.6 |
| *Vicia villosa* | Silica | 1 | 65 | 10.1 | 114 | 564.4 | 1.94 | 2.08 | 6396 | 5.7 |
| *Vicia villosa* | Silica | 2 | 50 | 10.1 | 53 | 262.4 | 1.99 | 2.13 | 6064 | 5.7 |
| *Vicia villosa* | Silica | 2 | 55 | 10.1 | 11.8 | 58.4 | 2.07 | 2.16 | 5233 | 3.5 |
| *Vicia villosa* | Silica | 2 | 60 | 10.1 | 32.1 | 158.9 | 2.03 | 2.14 | 6167 | 5.6 |
| *Vicia villosa* | Silica | 2 | 65 | 10.1 | 111 | 549.5 | 1.77 | 2.06 | 6076 | 5.7 |
| *Vicia villosa* | Silica | 18–20 | 50 | 10.1 | 131 | 648.5 | 2.03 | 2.09 | 4088 | 4 |
| *Vicia villosa* | Silica | 18–20 | 55 | 10.1 | 50.8 | 251.5 | 1.92 | 2.1 | 4695 | 4.9 |
| *Vicia villosa* | Silica | 18–20 | 60 | 10.1 | 40.2 | 199 | 1.93 | 2.13 | 4660 | 4.4 |
| *Vicia villosa* | Silica | 18–20 | 65 | 10.1 | 62.4 | 308.9 | 1.88 | 2.14 | 3771 | 3.9 |

^a^Tissue preservation type: Herb. = herbarium specimen, Silica = silica gel–preserved.

^b^The measure of DNA concentration from the Qubit.

^c^The amount of DNA recovered per tissue input weight; used to standardize measurements.

^d^260/230 is the spectral ratio that measures the ratio of DNA to secondary metabolites.

^e^260/280 is the spectral ratio that measures the ratio of DNA to proteins.

^f^The average fragment length between 100 and 60,000 bp as estimated with the Agilent Tapestation.

^g^DIN is the DNA integrity number that accounts for the quality and quantity of the DNA as measured in the Tapestation.
