# Supplementary material for: Optimizing the lysis step in CTAB DNA extractions of silica‐dried and herbarium leaf tissues
Source: Appl Plant Sci. 2023 May 27;11(3):e11522. doi: 10.1002/aps3.11522 (PMC10278933; doi:10.1002/aps3.11522)

DOI 10.1002/aps3.11522

APPENDIX S5. Effect of differences in incubation temperature for each of the four taxonomic groups on the (A) A_{260}/A_{230} ratio, (B) A_{260}/A_{280} ratio, (C) log-transformed average fragment length, and (D) log-transformed total amount of DNA given the input leaf tissue weight.

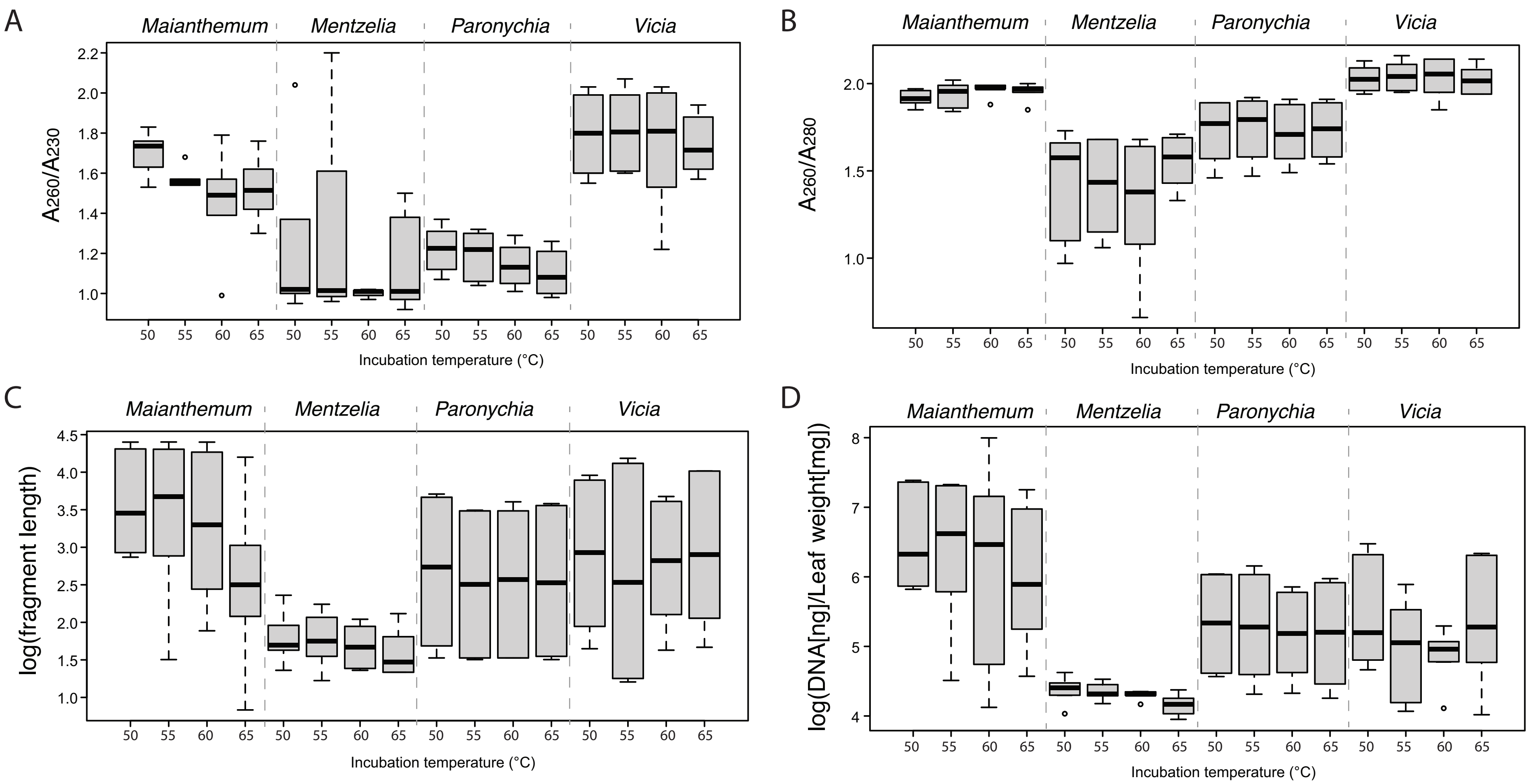

Supplement: Supplementary file 5 — Appendix S5. Effect of differences in incubation temperature for each of the four taxonomic groups on the (A) A260/A230 ratio, (B) A260/A280 ratio, (C) log‐transformed average fragment length, and (D) log‐transformed total amount of DNA given the input leaf tissue weight. [file APS3-11-e11522-s003.pdf]
